# Precision Medicine for Apical Lesions and Peri-Endo Combined Lesions Based on Transfer Learning Using Periapical Radiographs

**DOI:** 10.3390/bioengineering11090877

**Published:** 2024-08-29

**Authors:** Pei-Yi Wu, Yi-Cheng Mao, Yuan-Jin Lin, Xin-Hua Li, Li-Tzu Ku, Kuo-Chen Li, Chiung-An Chen, Tsung-Yi Chen, Shih-Lun Chen, Wei-Chen Tu, Patricia Angela R. Abu

**Affiliations:** 1 Department of General Dentistry, Taoyuan Chang Gung Memorial Hospital, Taoyuan City 32023, Taiwan; q384@cgmh.org.tw; 2 Department of Operative Dentistry, Taoyuan Chang Gung Memorial Hospital, Taoyuan City 32023, Taiwan; louiszzzzz@cgmh.org.tw; 3 Department of Program on Semiconductor Manufacturing Technology, Academy of Innovative Semiconductor and Sustainable Manufacturing, National Cheng Kung University, Tainan City 701401, Taiwan; m28121562@gs.ncku.edu.tw; 4 Department of Information Management, Chung Yuan Christian University, Taoyuan City 32023, Taiwan; s11044106@cycu.edu.tw (X.-H.L.); s11044114@cycu.edu.tw (L.-T.K.); 5 Department of Electrical Engineering, Ming Chi University of Technology, New Taipei City 243303, Taiwan; joannechen@mail.mcut.edu.tw; 6 Department of Electronic Engineering, Feng Chia University, Taichung City 40724, Taiwan; 7 Department of Electronic Engineering, Chung Yuan Christian University, Taoyuan City 32023, Taiwan; chrischen@cycu.edu.tw; 8 Department of Electrical Engineering, National Cheng Kung University, Tainan City 701401, Taiwan; wctu@gs.ncku.edu.tw; 9 Ateneo Laboratory for Intelligent Visual Environments, Department of Information Systems and Computer Science, Ateneo de Manila University, Quezon City 1108, Philippines; pabu@ateneo.edu

**Keywords:** apical lesion, peri-endo combined lesion, image segmentation, CNN, YOLOv8-OBB

## Abstract

An apical lesion is caused by bacteria invading the tooth apex through caries. Periodontal disease is caused by plaque accumulation. Peri-endo combined lesions include both diseases and significantly affect dental prognosis. The lack of clear symptoms in the early stages of onset makes diagnosis challenging, and delayed treatment can lead to the spread of symptoms. Early infection detection is crucial for preventing complications. PAs used as the database were provided by Chang Gung Memorial Medical Center, Taoyuan, Taiwan, with permission from the Institutional Review Board (IRB): 02002030B0. The tooth apex image enhancement method is a new technology in PA detection. This image enhancement method is used with convolutional neural networks (CNN) to classify apical lesions, peri-endo combined lesions, and asymptomatic cases, and to compare with You Only Look Once-v8-Oriented Bounding Box (YOLOv8-OBB) disease detection results. The contributions lie in the utilization of database augmentation and adaptive histogram equalization on individual tooth images, achieving the highest comprehensive validation accuracy of 95.23% with the ConvNextv2 model. Furthermore, the CNN outperformed YOLOv8 in identifying apical lesions, achieving an F1-Score of 92.45%. For the classification of peri-endo combined lesions, CNN attained the highest F1-Score of 96.49%, whereas YOLOv8 scored 88.49%.

## 1. Introduction

Apical lesions are primarily caused by bacteria and microorganisms entering the apex through dental caries, trauma, overheating, malocclusion, and other ways. Due to the small capacity of the pulp chamber, initial inflammation of the pulp spreads along the canals to the apex, forming a pathogenic mechanism with the root inflammation. The lack of obvious pain symptoms in early stages makes it difficult to detect until the pulp has already been necrotic. Periodontal disease results from bacteria in plaque. Poor oral hygiene leads to the accumulation of bacteria in the gingiva and alveolar bone. In mild cases, it is primarily related to the calculus from saliva calcification, leading to inflammation of the surrounding tissues. In severe cases, inflammation worsens and erodes from top to bottom, eventually separating the gingiva and tooth roots. Apical lesion is the destruction of the tooth’s inner part, while periodontal disease is from the outside to the inside. When these two infections progress simultaneously and connect, it is a peri-endo combined lesion, which means that the prognosis of this symptom will be significantly reduced. According to the degree of damage and the ability to repair, the infections could be categorized into primary pulpal infection, primary periodontal infection, independent manifestations of primary pulpal and periodontal infections, and combined manifestations of both primary pulpal and periodontal infections [1]. Therefore, dentists must carefully evaluate the area below the crown–root junction to assess symptoms.

The differences in access to oral health services in the United States are largely due to socioeconomic status. When a person’s overall health is lacking, oral diseases can worsen existing conditions and lead to more complications [2]. This is why improving service quality, accessibility, and patient-centered care across all levels is such an important goal. Currently, medical diagnosis relies on manual identification, imaging diagnosis, vitality tests, and past clinical experience. Dentists must pay attention to the presence, absence, and location of any swelling or drainage. Besides necessary root canal treatment, patients may also require periodontal treatment. However, the success rate of tooth retention cannot be guaranteed after treatment [3]. During this process, different dentists have different perspectives and experiences, and adding various symptoms involving pulpal, periodontal, or mixed lesions further increases the complexity. Untreated endodontic infections can lead to pocket formation, bone loss, calculus deposition, osteoclastic activity, and bone resorption. These infections can also impair wound healing and worsen periodontal disease [4]. Based on the above, this research aims to establish a medical image recognition system that adopts image-processing techniques and deep learning models to analyze PAs and classify them into three dental conditions: 0—asymptomatic, 1—apical lesion, and 2—peri-endo combined lesion. The purpose is to achieve intelligent diagnosis, facilitating early detection of potential problems and enabling early treatment and prevention. It significantly reduces dentists’ time spent on complicated tasks, allowing them to focus more on providing deeper patient care to improve clinical efficiency.

Numerous studies are underway in the medical field, where the integration of artificial intelligence (AI) and medical imaging has significantly advanced dental radiology. Integrating CNN and other deep learning techniques can enhance diagnostic accuracy and optimize clinical workflows. Dental panoramic radiographs [5], PAs [6], and bitewing radiographs [7] are essential diagnostic tools in dentistry, providing comprehensive insights into various dental conditions. Automated detection systems are gradually replacing traditional manual interpretation methods. For example, the U-Net CNN algorithm is used for segmentation in DPR, achieving an F1-score of 0.828 [8]. Faster R-CNN technology is successfully used to detect seven types of dental conditions on DPR, such as apical lesions and implants, achieving an accuracy rate of 94.18% [9]. The model architecture based on Mask-RCNN and MobileNet-v2 detects and locates five types of periapical lesions with an accuracy of 94%, a mean average precision of 85%, and a mean intersection over a union of 71.0% [10]. These systems accurately enhance dentists’ diagnostic capabilities. Furthermore, the introduction of transfer learning has facilitated highly specialized CNN models for dental tasks, such as detecting retained roots [11], apical lesions [12], missing teeth [13], and restorations [7]. By using pre-trained CNN architectures, researchers have obtained outstanding performance and surpassed traditional methods. For instance, the YOLOv2 model achieves an accuracy of 89.3% in identifying implant positions, while the AlexNet model demonstrates an accuracy of 90.4% in evaluating the damage caused by periodontitis around implants [14].

In addition to the selection and training of models, image enhancement and segmentation techniques are also crucial, as PA films can possibly have a significant amount of noise. In addition to this, the objects are apical lesions, peri-endo combined lesions, and asymptomatic cases. It is challenging for us to effectively and precisely enhance the symptom area and ensure that the improved features are captured and learned by the model. Sharpening, histogram equalization [15], and flat-field correction are employed to enhance images. Image segmentation is used to accurately label the position and number of each tooth, resulting in a 92.78% accuracy rate. This research adopts various image-processing techniques, including grayscale conversion [16], Gaussian high-pass filter [17,18], adaptive histogram equalization [19], linear transformation, flat-field correction, and negative film effect. These methods improve image quality and enhance features, thereby increasing the accuracy of subsequent training. The YOLOv8 model [20] not only detects the position of individual teeth, but also precisely handles teeth at irregular angles with OBB technology [21]. This adaptation facilitates more efficient and accurate object detection and subsequent image cropping, which are crucial for building the CNN training dataset. In this study, several CNN models are chosen to classify different diseases. A control group used YOLOv8 for object detection, and by comparing and evaluating these models, the one with the best generalization capability and highest accuracy was selected for this research. The purpose of using multiple models is to identify the best experimental method.

The purpose of using multiple models is to identify the best experimental method. This study aims to use machine learning technology to assist doctors throughout the entire treatment process of apical lesions and peri-endo combined lesions. This includes early detection and assessment of symptoms to avoid delayed treatment, mid-term assistance to prevent deterioration due to incomplete treatment, and final confirmation at a later stage to avoid recurrence. The use of multiple models is a deliberate strategy to rigorously identify the most effective experimental approach. This research integrates advanced algorithms for early detection; real-time support during treatment; and thorough follow-up to prevent delays, deterioration, and recurrence. This comprehensive use of machine learning enhances diagnostic precision and ensures effective, continuous care.

## 2. Materials and Methods

**Figure 1:**
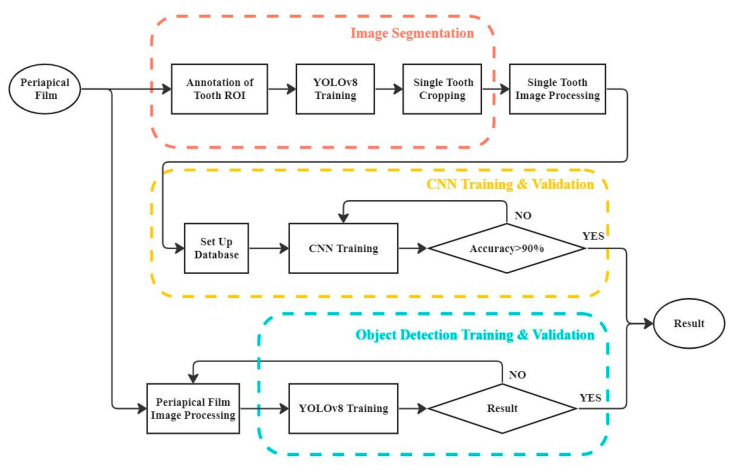
Research flowchart.

To adequately detect the symptoms apical lesions and peri-endo combined lesions, the flowchart which is shown in Figure 1 encompasses comprehensive steps from image segmentation to image processing, followed by CNN and object detection training and validation, to ensure accurate symptom classification by the model. This research received approval from the Chang Gung Medical Foundation Institutional Review Board, with the IRB number 02002030B0. The image database used in this study was annotated by dentists with over five years of experience and divided into training and validation datasets.

### 2.1. Image Segmentation

The PA images provide detailed views of tooth roots and surrounding periodontal structures, making them crucial for diagnosing apical lesions and peri-endo combined lesions, and each PA film contains 2 to 5 teeth. For CNN-based single-tooth pathology image classification, this study first processes the images to isolate individual teeth, allowing the CNN to accurately classify tooth pathologies.

#### 2.1.1. Tooth Annotation

**Figure 2:**
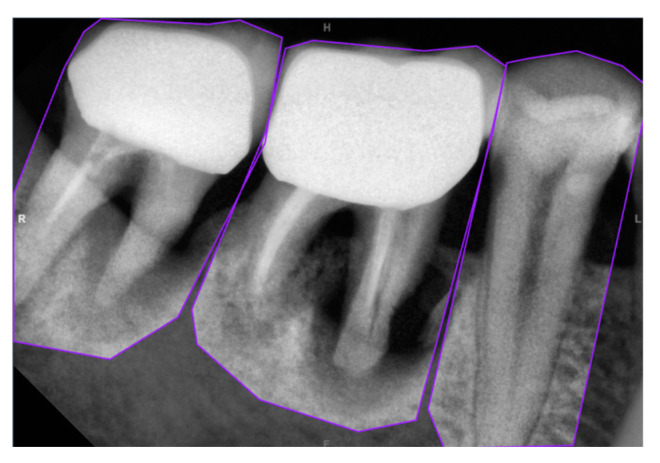
Manual annotation using Roboflow’s polygon tool.

This study uses Roboflow as the annotation tool, leveraging its polygon tool to accurately mark the actual shape and arrangement of teeth in each image, as shown in Figure 2. This process helps to minimize the annotation of non-target areas, providing more explicit benchmarks and data for model learning.

#### 2.1.2. YOLOv8 OBB Model Training

Several studies have focused on designing more complex object detection networks like SSD and YOLO. The object detection model is used to identify individual teeth and record their coordinates, followed by image segmentation using an algorithm specifically designed for this study. YOLO has an advantage in single-instance detection due to its efficiency and comprehensive feature capture. YOLOv8’s OBB excels in handling complex tooth arrangements and adapting to various angles.

#### 2.1.3. Single-Tooth Cropping

Single-tooth cropping from PA images is crucial for focused diagnosis and treatment planning. This process involves isolating individual teeth from a PA radiograph to enhance the accuracy and effectiveness of dental assessments by employing advanced image-processing techniques, such as deep learning algorithms. This minimizes the interference from surrounding structures, enabling a clearer view of the tooth’s condition.

A. Image Rotation

**Figure 3:**
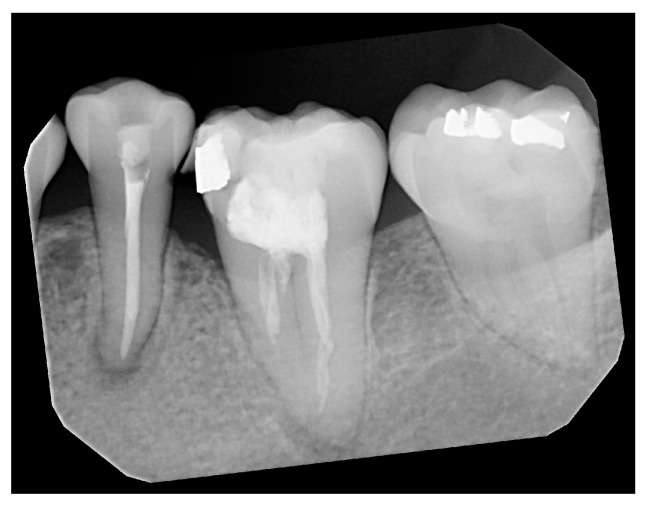
The first tooth rotated to a horizontal 0-degree image.

The trained YOLO optimal model is used for single-tooth detection in PA images, obtaining the OBB information of each tooth region, including position, size, and angle. Next, each detected tooth region undergoes individual processing steps. The rotation angle of each tooth region is calculated with the center of the PA image designated as the rotation center. The image is rotated to a horizontal position with a 0-degree angle, as shown in Figure 3. This ensures that the cropped image is a non-tilted rectangle, avoiding the loss of essential image parts due to the original skew angle.

B. Coordinate Point Rotation

The four corner coordinates of the tooth also need to be rotated to the same angle for precise image cropping with consistent rotation. Initially, the four corner coordinates of each tooth’s bounding box are obtained based on the tooth’s position. These coordinates are rotated using a rotation matrix, as shown in Equation (1). The rotation matrix rotates a point in the xy-plane counterclockwise by an angle θ around the origin.

(1) $$Rv\;=\;\left[cos\theta \;-\;sin\theta \;sin\theta \;cos\theta \right]\left[x\;y\right]\;=\;\left[x\;cos\theta \;-\;y\;sin\theta \;x\;sin\theta \;+\;y\;cos\theta \right]$$

C. Single-Tooth Cropping and Cropping Area Expansion

**Figure 4:**
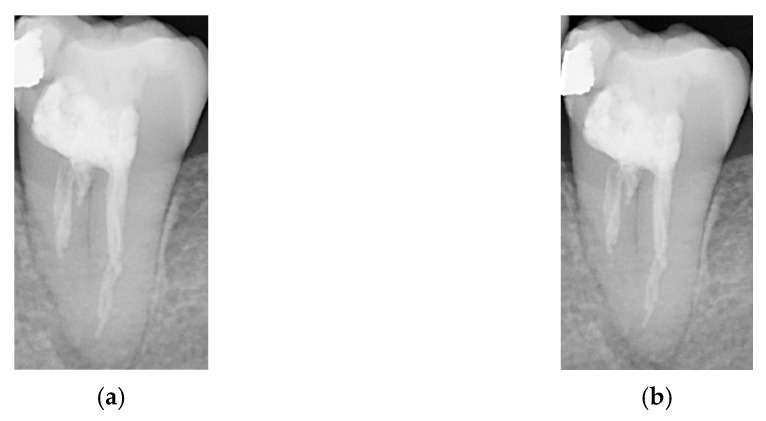
Image expansion. (**a**) Original cropped image. (**b**) Cropped image expanded by 20 pixels horizontally and 40 pixels vertically.

Each tooth is cropped to obtain individual tooth images, which is crucial for diagnosing apical lesions and peri-endo combined lesions by focusing on apical and periodontal edge features. However, the original detection range might accidentally cut off parts of the apex or periodontal area. To address this, the study expanded the detected tooth area before cropping, allowing more image features to be captured. Figure 4 compares the images before and after this expansion. This approach helps to determine the optimal cutting range, maximizing feature extraction while minimizing noise.

### 2.2. Image Processing

This study uses several image enhancement methods to conduct comparisons and cross-experiments, including grayscale transform, Gaussian high-pass filters, adaptive histogram equalization, linear transformation, flat-field correction, and negative film effect. The goal is to find the best combination, providing clear and high-quality images to help the system analyze and identify disease symptoms more effectively, thus improving the accuracy of the model.

#### 2.2.1. Grayscale

The first step in image processing is converting the original color images into grayscale images which only contain luminance information. In this study, the PA image database is not a grayscale image database. This method ensures that subsequent processing is based on single-channel grayscale data, simplifying the complex stereoscopic data while retaining the basic structure and features of the images, thus providing a solid foundation for subsequent preprocessing steps.

#### 2.2.2. Gaussian High-Pass Filter

**Figure 5:**
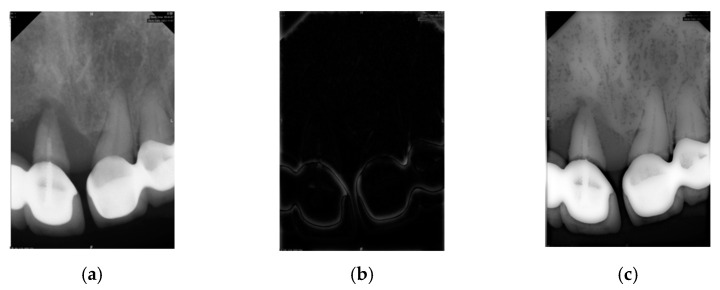
The Gaussian high-pass filter result. (**a**) The original image. (**b**) The result of the Gaussian high-pass filter. (**c**) The result of (**a**) minus (**b**).

The Gaussian high-pass filter is a frequency domain technique used in image processing to sharpen images and enhance details by filtering out low-frequency parts and retaining high-frequency parts. Based on the Gaussian function, the filter’s sharpness is controlled by adjusting the cutoff frequency *D*_0_, affecting the emphasis on high frequencies, as shown in Equation (2). The filter result is shown in Figure 5.

(2) $$H\left(u,v\right)=1-e^{-D^{2}(u,v)/2{D_{0}}^{2}}$$

#### 2.2.3. Adaptive Histogram Equalization

**Figure 6:**
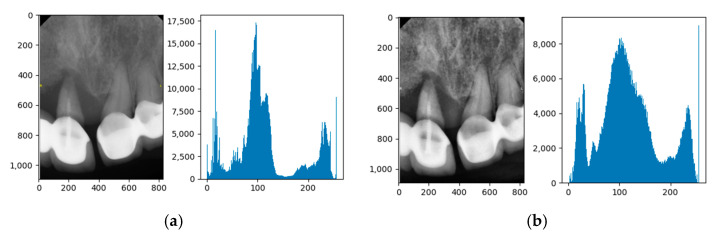
The adaptive histogram equalization. (**a**) Original image and histogram. (**b**) Enhanced image and histogram after adaptive histogram equalization.

Adaptive histogram equalization enhances image contrast by making local adjustments based on brightness variations in different regions. The process involves dividing the image into equally sized regions, equalizing the histogram of each region to distribute pixel values evenly, and then recombining these regions to produce the final enhanced image, as shown in Figure 6.

#### 2.2.4. Flat-Field Correction

**Figure 7:**
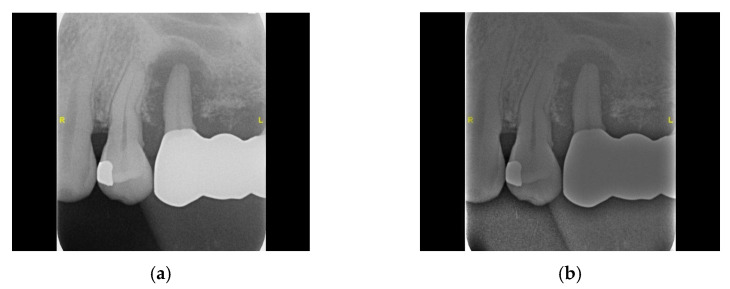
The flat-field correction result. (**a**) Original image. (**b**) Flat-field correction image.

The flat-field correction technique can eliminate or reduce deformations or artifacts caused by the shooting angle, bringing the images closer to the actual dental morphology. This enhances image quality and detail, such as the texture and edge contours of the tooth surface shown in Figure 7, and improves image contrast and color fidelity, enabling the model to observe dental features and make more precise judgments.

#### 2.2.5. Linear Transformation

**Figure 8:**
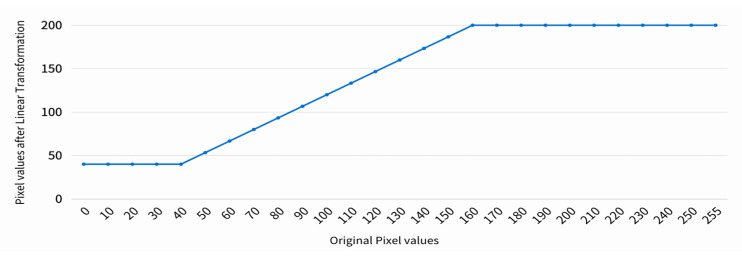
Linear transform model.

**Figure 9:**
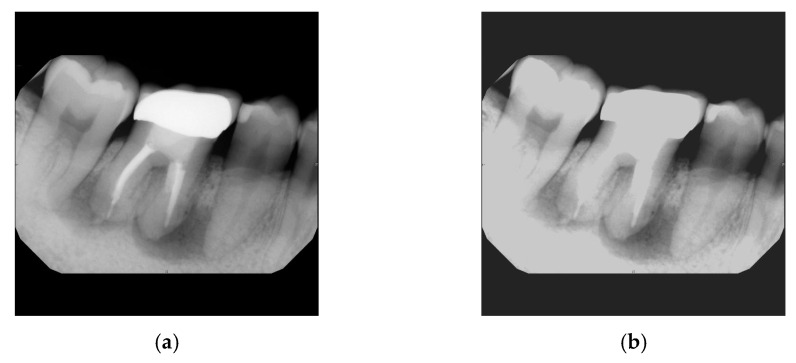
The result of linear transformation. (**a**) Original image. (**b**) Linear transformation image.

Linear transformation can effectively enhance image quality and readability, offering highly flexible adjustments to process pixel values within specific ranges according to different needs and purposes. In this study, the pixel values in the image are divided into three blocks: (1) below 40, (2) between 40 and 160, and (3) above 160. The pixel values in block 1 are uniformly set to 40. Block 2 is transformed to new pixel values not exceeding 160 according to Equation (3). Among this function, x is the original pixel value and y is the pixel value after linear transformation. Block 3 is uniformly set to 200. This setup adjusts the extremely dark and bright parts to enhance the overall visual consistency of the image. In addition, local contrast enhancement is performed on the image area in block 2 to make details more transparent or more prominent while limiting the values so that they do not exceed 160 to avoid overexposure or oversaturation. The linear transformation result is shown in Figure 8, and enhanced results are shown in Figure 9.

(3) $$y=\frac{4}{3}x-\frac{40}{3}$$

#### 2.2.6. Negative Film Effect

**Figure 10:**
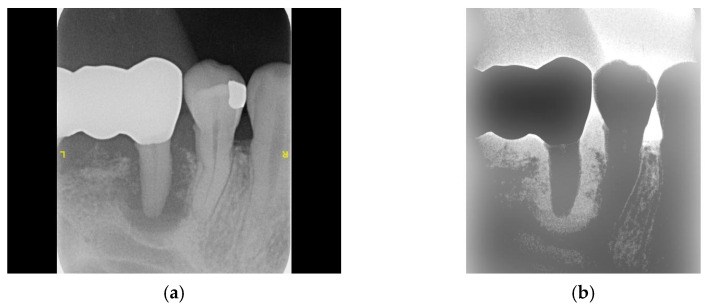
The result of negative film effect. (**a**) Original image. (**b**) Negative film effect image.

The negative film effect is used to invert the colors and brightness of the original image, making details and contrast stand out more clearly. This makes it easier to see the extent and spread of apical and periodontal erosion, especially the edge contours. The original PA image is first converted to grayscale, with pixel values ranging from 0 (black) to 255 (white). Then, symptom enhancement is applied by subtracting the grayscale values from 255, which flips black to white and white to black. The results are shown in Figure 10.

### 2.3. CNN Training and Validation

In medical imaging automatic detection systems, deep learning techniques are widely adopted due to their excellent feature learning capabilities. CNNs capture the spatial structures of images through convolutional layers, effectively handling local features and improving parameter efficiency through weight sharing. Most medical research employs CNN models, such as the study on dental detection in reference [22], which used a custom CNN architecture to achieve an accuracy of 93.04%. This study employs six well-known CNN models, aiming to identify the most accurate model through evaluation and comparison, to provide more reliable clinical symptom recognition. The study uses an Nvidia GeForce RTX 3070 GPU to accelerate model training, with specific hardware and software platforms detailed in Table 1. To optimize model performance to the maximum extent, this study implements the following strategies. The first is hyperparameter tuning. Systematic and combinatorial hyperparameter adjustments, such as learning rate and batch size, will optimize the model’s training process. This step aims to improve the model’s convergence speed and performance, making it more suitable for recognition tasks for specific symptoms. The second is overfitting prevention. Dropout is a commonly used regularization technique in deep learning. Dropout randomly drops some neurons and their connections during training, ensuring that the model uses different subsets for each training iteration. This prevents the network from relying on any single neuron. And the last is metric evaluation, paying high attention to loss function, accuracy, precision, recall, and F1-score during the model-training process. These metrics provide in-depth insights into the model’s performance in various aspects, helping to analyze the model’s strengths and limitations comprehensively.

#### 2.3.1. CNN Architecture

This study adopts six CNN models: AlexNet, Places365-GoogLeNet, VGG-16, ResNet50, GoogLeNet, and ConvNeXt-v2. ConvNeXt-v2 has a powerful detection ability because it combines ConvNeXt, Global Response Normalization, and fully convolutional masked auto-encoder (FCMAE). GRN enhances feature competition between channels, including contrast and selectivity. FCMAE is a transformer specifically tuned for ConvNeXt-v2, and it can randomly mask the original image and let the model learn based on the remaining context. This architecture effectively alleviates the feature collapse, maintains feature diversity during training, and improves accuracy. Taking AlexNet as an example, its architecture is detailed in Table 2. The input size is set to 227 × 227 × 3 pixels. Given that this study’s image classification task involves only three categories, the output size of the last fully connected layer is adjusted from the original 1000 to 3 to match the classification task requirements. A dropout probability of 0.7 is set to reduce model overfitting effectively.

#### 2.3.2. Hyperparameter

Key hyperparameters in deep learning model training include Initial Learning Rate, Max Epoch, and Mini Batch Size. The Initial Learning Rate, which determines the speed of parameter updates, is optimized to 0.0001 after experimenting with rates from 0.1 to 0.00001. Max Epoch settings are adjusted for different CNN models to influence learning extent. Mini Batch Size, affecting training speed and generalization, is set between 4 and 64, with 16 found to be optimal. These adjustments aim to minimize the loss function on the test dataset, improving model performance and generalization. This study applies the best hyperparameter settings from tuning AlexNet to other CNN models to ensure fair comparisons. While unified settings help to avoid unfair comparisons, they might cause overfitting in some models. Therefore, while keeping parameters like the Initial Learning Rate, Mini Batch Size, Learning Rate Drop Factor, and Learning Rate Drop Period consistent, Max Epoch is adjusted to prevent overfitting. The CNN model’s hyperparameter settings are shown in Table 3.

#### 2.3.3. Training and Validation

The dataset is split into 80% training and 20% validation sets to ensure the independence and credibility of model training and validation, as shown in Table 4. Considering the insufficient original data, this study adopts data augmentation to expand the data volume by fourfold. The augmented data quantities are listed in Table 5. Data augmentation mainly involves horizontal and vertical flipping to increase the dataset and strengthen the model. To ensure that the model performs well with new, unknown data, researchers balance the numbers of asymptomatic and symptomatic teeth. This helps to prevent the model from overly biasing any category, thereby enhancing overall performance and adaptability.

This study assesses the model’s performance using four metrics after training: accuracy, precision, recall, and F1-score, as detailed in Equations (4)–(7). These metrics comprehensively assess the model’s prediction ability, recognition capability for different categories, and overall performance level, providing an objective evaluation of the model’s strengths and weaknesses. By using these evaluation standards, we ensure that the trained model is reliable and performs well in practical applications.

(4) $$Accuracy\;=\;\frac{TP+TN}{TP+TN+FP+FN}$$

(5) $$Precision=\frac{TP}{TP+FP}$$

(6) $$Recall=\frac{TP}{TP+FN}$$

(7) $$F1\;Score=2\times \frac{precision\times recall}{precision+recall}$$

### 2.4. Object Detection Training and Validation

This study conducts experiments on YOLOv8 using two models, YOLOv8n and YOLOv8s-OBB, to compare the imported original PA and to carry out object detection and classify several teeth in the image, thus optimizing the cumbersomeness of the training process, omitting the steps of cutting a single tooth, and classifying the training set. In addition, researchers hope to use the advantages of YOLOv8 and image enhancement technology to find a training method with high accuracy. The model hyperparameter reference is shown in Table 6.

**Figure 11:**
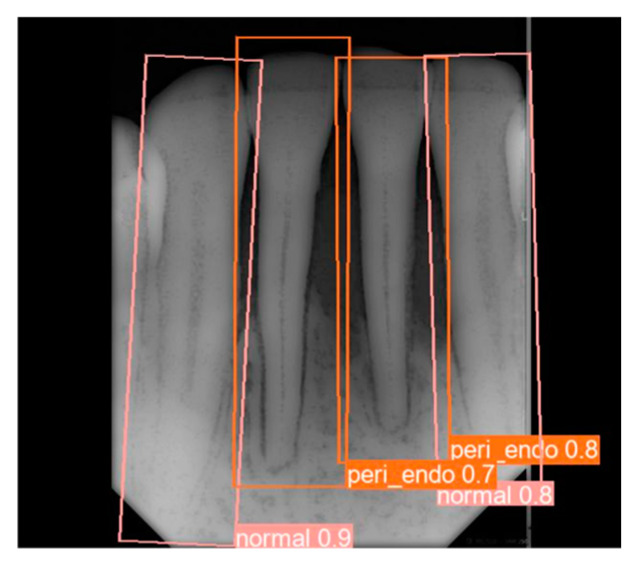
The disease prediction results with YOLOv8 OBB.

Apart from basic object detection, YOLOv8 offers three notable advantages: it features new convolutional layers, replacing the c3 module with the c2f module, modifying various convolutions for greater efficiency, and using a Decoupled head while removing the objectness branch. It also supports anchor-free detection, eliminating the need for manual anchor boxes by directly predicting the object’s center and enhancing flexibility and efficiency. YOLOv8-OBB is crucial for accurately detecting teeth with varying orientations and angles, improving detection and classification accuracy without the need for repeated image preprocessing steps, thus streamlining the process and enhancing experimental efficiency. The prediction results are shown in Figure 11.

## 3. Results

This section discusses the outcomes of YOLO detection and image segmentation, image classification, and object detection.

### 3.1. YOLO Detection and Image Segmentation

**Figure 12:**
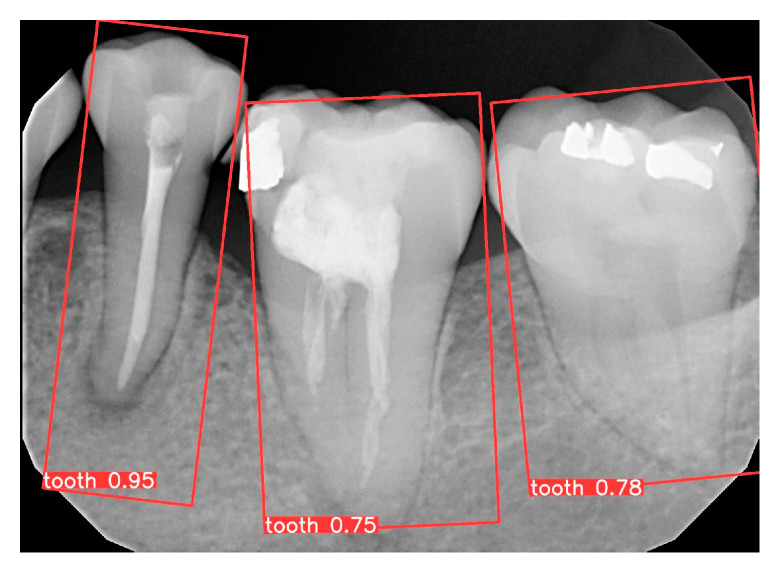
Single-tooth prediction results.

The YOLOv8 OBB model was employed to identify individual teeth and record their coordinates. During the experimental phase, 147 PA images and their corresponding annotations were prepared and divided into training, validation, and test sets in a 7:2:1 ratio. Specifically, 103 images were used for training, 29 for validation, and 15 for testing. The object detection performance is illustrated in Figure 12, where three teeth were effectively detected in the periapical images. The model achieved a precision of 93.9%, a recall of 97.7%, and a mean average precision (mAP50) of 97.3%. This stage achieved precise tooth detection while preserving complete pathological features, providing a reliable foundation for CNN classification.

### 3.2. CNN Training

**Figure 13:**
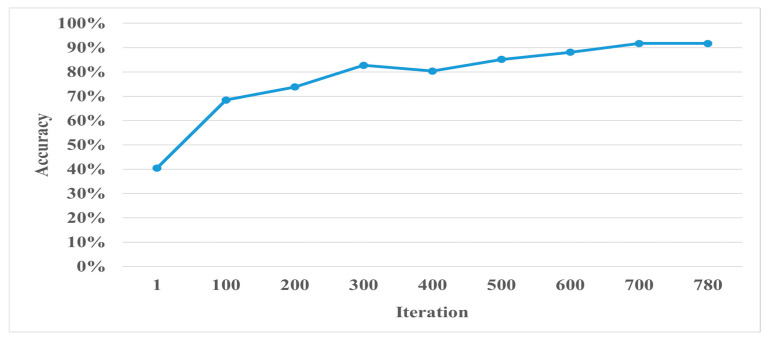
Validation accuracy during the training process of the Places365-GoogLeNet model.

**Figure 14:**
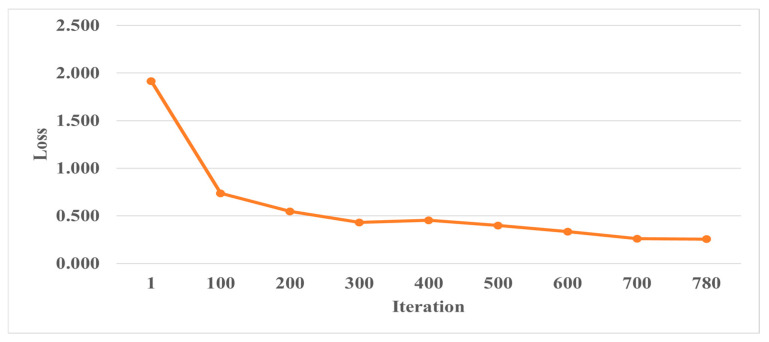
Validation loss function during the training process of the Places365-GoogLeNet model.

During the CNN model training process, it is essential to monitor the accuracy and loss function of the validation set. If the accuracy of the training set continues improving while the validation set accuracy stays the same or decreases, it could indicate that the model is overfitting. To address this, regularization methods are needed to prevent overfitting. For example, in the case of Places365-GoogLeNet, the validation accuracy and loss function are shown in Figure 13 and Figure 14. By analyzing these metrics, training strategies and parameters can be adjusted to improve accuracy.

**Figure 15:**
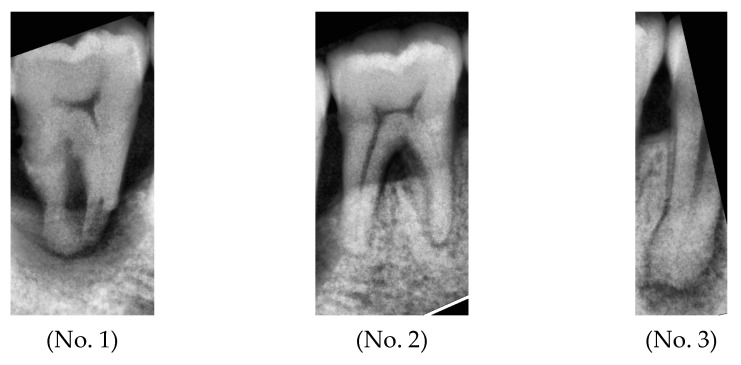
After adaptive histogram equalization, segmentation into multiple single-tooth images is conducted (numbered from left to right).

This study used YOLOv8 for image segmentation and performed adaptive histogram equalization, as shown in Figure 15. The processed images were input into the trained AlexNet model, comparing the obtained validation results with ground truth values and recording the corresponding prediction probabilities, as detailed in Table 7.

To detect apical and peri-endo combined lesions, this study explored various image processing methods and identified effective models through multiple combination experiments. Four aspects were analyzed: black padding, data augmentation, cropping range expansion, and image enhancement. Six CNN models were tested across these aspects to find the best combination.

1. Aspect 1: Black Padding

Most CNN models require square images as input to simplify the model design, facilitate feature extraction, and simplify internal computations, improving training efficiency and accuracy. Since tooth shapes and sizes vary, fitting a 1:1 square size is challenging. Therefore, two common methods were adopted for image processing: transforming individual teeth into a 1:1 size or placing teeth centrally while padding the sides with black pixels. After processing images with these two methods and training the CNN models, the results, as shown in Table 8, indicated that models using black pixel padding performed better, with the validation accuracy improving by 1.76–9.52%. For example, ConvNeXtv2’s accuracy improved from 76.19% to 85.71%, and GoogLeNet’s accuracy increased from 80.95% to 85.71%. These improvements highlight the effectiveness of black pixel padding in enhancing model performance.

2. Aspect 2: Data Augmentation

Considering the limited data in this study, data augmentation is essential for enhancing model robustness and generalization ability. This study applied data augmentation to expand the original dataset by fourfold. The experimental results are shown in Table 9 indicating that the validation accuracy increased by 1.19% to 14.88% across different models. For instance, AlexNet’s accuracy improved from 83.33% to 88.69%, and Places365-GoogLeNet’s accuracy increased from 80.95% to 88.69%. These results highlight the effectiveness of data augmentation in scenarios with limited samples, validating its role in improving model performance.

3. Aspect 3: Expanding the Cropping Range

Researchers aimed to expand the tooth region to capture more useful diagnostic features, increasing accuracy without introducing irrelevant features. This study conducted four trials: no expansion, horizontal expansion by 20 pixels, vertical expansion by 40 pixels, and both horizontal and vertical expansions. The results in Table 10 show that different models responded differently to these expansions. For example, Places365-GoogLeNet achieved its highest accuracy (91.67%) with vertical expansion by 40 pixels, while ConvNextv2 reached 91.07% accuracy with both expansions. This highlights the need for tailored strategies depending on the specific CNN model.

4. Aspect 4: Image Enhancement

Researchers explored the impact of different image enhancement methods on model training, specifically testing combinations of Gaussian high-pass filters and adaptive histogram equalization. These enhancement techniques significantly improved model validation accuracy, with increases ranging from 2.38% to 7.73%, as shown in Table 11. Both using the Gaussian high-pass filter alone and applying adaptive histogram equalization after Gaussian high-pass filtering proved to be effective strategies. For instance, ConvNeXtv2’s accuracy improved to 95.23% with adaptive histogram equalization, demonstrating the effectiveness of these image enhancement methods in boosting model performance. The ConvNextv2 model utilized black padding, data augmentation, and adaptive histogram equalization and achieved a validation accuracy of 95.23%, making it the best model in this study. The confusion matrix for this model is shown in Table 12. Comparisons with other studies on apical lesion detection demonstrated that this method significantly outperformed others in precision and F1-score, as detailed in Table 13. The ConvNextv2 model, evaluated for classifying dental conditions into normal, apical lesion, and peri-endo combined lesion, achieved an overall validation accuracy of 95.23%. The model showed exceptional performance with high precision (93.33%) and recall (99.75%) for normal teeth, resulting in a strong F1-score of 96.55%. For apical lesions, the precision was also high at 98.00%, though recall was lower at 87.50%, leading to an F1-score of 92.45%. The model excelled in identifying peri-endo combined lesions, with a precision of 94.82% and recall of 98.21%, yielding an F1-score of 96.49%.

### 3.3. YOLOv8

This study used YOLOv8 object detection as a comparison group. Compared to the CNN model’s image recognition, YOLOv8 optimized preparatory steps and improved validation accuracy through data augmentation and image processing combinations, resulting in high efficiency. In YOLO models, two critical metrics are mAP50 and validation accuracy. A mAP50 value close to 1 indicates that the predicted bounding boxes overlap with the ground truth boxes by more than 50%, reflecting a good object detection capability. The number of objects in each image category is crucial. Table 14 shows the number of instances in the original training and validation sets, with an 8:2 ratio.

To achieve better accuracy and model generalization, data augmentation was applied to the original dataset, including horizontal, vertical, and 90-degree rotations. This effectively increased the diversity of training data, helping the model learn various tooth angles, directions, and features, thereby enhancing overall detection and prediction performance. As shown in Table 15, data augmentation improved accuracy by 9.7%, and mAP50 increased by 0.035 to 0.136, significantly strengthening the model’s ability to identify candidate areas for normal and apical lesion categories.

Building on data augmentation, this study explored the performance of YOLOv8 object detection on PA images preprocessed with various image-processing techniques, as detailed in Table 16. Linear transformation with adaptive histogram equalization achieved an overall accuracy of 85.16%; flat-field correction with adaptive histogram equalization reached 87.79%; and the combination of a Gaussian high-pass filter with a negative film effect performed the best, with an overall accuracy of 92.13%. These findings highlight the importance of selecting appropriate image-processing techniques to enhance model accuracy and detection capabilities.

Three effective combination methods were identified: increasing contrast and balancing image quality through two combinations—linear transformation plus adaptive histogram equalization and flat-field correction plus adaptive histogram equalization—as well as denoising, enhancing edge contours and details by using a Gaussian high-pass filter plus the negative image effect. The combination of a Gaussian high-pass filter and the negative film effect was the best, achieving an accuracy of 92.13% and an increase of 7.43%, with mAP50 consistently above 0.9 for all categories and overall, as shown in Table 17. A comparison with other studies using YOLO models is provided in Table 18.

## 4. Discussion

The findings of this study demonstrate significant advancements in the detection of apical lesions and peri-endo combined lesions using deep learning models, particularly CNNs and YOLOv8 object detection. This study is the first to use deep learning to assist in the diagnosis of peri-endo combined lesions, achieving a recall rate of up to 91.7%. Through the implementation of various image-processing techniques—such as black padding, data augmentation, cropping range expansion, and image enhancement—the system achieved significant improvements in model accuracy and robustness. Using various image-processing techniques and data augmentation strategies significantly boosted model accuracy and generalization. For instance, applying black padding improved the validation accuracy by 1.76% to 9.52%, showing how effective it is in standardizing image sizes and enhancing performance. Expanding the dataset through data augmentation increased the accuracy by 1.19% to 14.88%, emphasizing its essential role in addressing small dataset limitations and boosting the model’s robustness. Expanding the cropping range of tooth regions captured more diagnostic features, improving accuracy by 0.59% to 3.57% across different models. Image enhancement techniques, such as Gaussian high-pass filters and adaptive histogram equalization, further boosted accuracy by 2.38% to 7.73%, with the combination of these methods proving to be particularly effective.

Comparing these research results with previous studies, the ConvNextv2 model achieved a validation accuracy of 95.23%, outperforming other model methods in precision and F1-score. For instance, the proposed method significantly outperformed the U-Net model used in apical lesion segmentation, which had an F1-score of 0.828, and the Faster R-CNN technology for various dental conditions detection, which achieved an accuracy of 94.18%. YOLOv8 was applied to PA images that were preprocessed using various image-processing techniques. Among these techniques, the combination of a Gaussian high-pass filter with a negative film effect yielded the highest overall accuracy of 92.13%, which led to a 7.43% improvement in validation accuracy. This approach consistently achieved high mAP50 values across different dental conditions, demonstrating its efficacy in improving detection capabilities. However, the study has limitations, including the potential for overfitting because of the limited number of samples in the database. Despite the limitations posed by the relatively small dataset, the findings underscore the potential of deep learning models in enhancing dental diagnostics. Future research should focus on expanding the dataset and refining preprocessing methods to further improve model performance and generalization. Additionally, developing standardized criteria for lesion severity, location, and spread will be crucial for improving the precision of automated detection systems.

## 5. Conclusions

This study offers valuable insights into how AI can be applied to medical imaging, helping to develop more reliable and efficient diagnostic tools in dentistry. The ConvNextv2 model stood out with a validation accuracy of 95.23%, outperforming other methods in both precision and F1-score. Additionally, using a Gaussian high-pass filter combined with the negative film effect in YOLOv8 led to the highest accuracy of 92.13%, highlighting the importance of choosing the right preprocessing techniques.

## Figures and Tables

**Table 1:** The hardware and software platform versions used in this study.

Hardware Platform	Version		
CPU	11 Gen Intel(R) Core(TM) i9-11900@2.50GHz		
GPU	NVIDIA GeForce RTX 3070 8G		
DRAM	32 GB		
Software Platform	Version	Software Platform	Version
MATLAB	R2023b	Python	3.11.8
Deep Network designer	R2023b	PyTorch	2.2.1 + cu121
Deep Learning Toolbox	23.2	CUDA	12.1

**Table 2:** AlexNet architecture.

Layer	Filters/Neuron	Filter Size	Stride	Padding	Size of Feature Map	Activation Functions
Input					227 × 227 × 3	
Conv 1	96	11 × 11	4		55 × 55 × 96	ReLU
MaxPool 1		3 × 3	2		27 × 27 × 96	
Conv 2	256	5 × 5	1	2	27 × 27 × 256	ReLU
MaxPool 2		3 × 3	2		13 × 13 × 256	
Conv 3	384	3 × 3	1	1	13 × 13 × 384	ReLU
Conv 4	384	3 × 3	1	1	13 × 13 × 384	ReLU
Conv 5	356	3 × 3	1	1	13 × 13 × 256	ReLU
MaxPool 3		3 × 3	2		6 × 6 × 256	
Dropout 1	Rate = 0.7				6 × 6 × 256	
Fc 1					4096	ReLU
Dropout 2	Rate = 0.7				4096	
Fc 2					4096	ReLU
Fc 3					3	Softmax

**Table 3:** CNN model hyperparameter settings.

Hyperparameter	Value	Hyper Parameter	Model	Value
Initial Learning Rate	0.0001	Max Epoch	AlexNet	30
Mini Batch Size	16		Places365-GoogLeNet	20
Learning Rate Drop Factor	0.1		VGG-16	6
Learning Rate Drop Period	10		ResNet50	10
Shuffle	Every-epoch		GoogLeNet	20
Validation Frequency	100		ConvNeXtv2_base	10

**Table 4:** CNN training set and validation set quantity.

Disease	Training Set	Validation Set	Total
Normal	53	14	67
Apical Lesion	53	14	67
Peri-endo Combined Lesion	53	14	67
Total	159	42	201

**Table 5:** The number of periapical images after data enhancement.

Disease	Original	Augmentation
Normal	67	268
Apical Lesion	67	268
Peri-endo Combined Lesion	67	268

**Table 6:** The hyperparameter value used in YOLOv8.

Hyperparameter	Value
Epoch	100
Batch	8
imgsize	640 × 640
lr0	0.01

**Table 7:** Comparison of validation results with ground truth.

Number	No. 1	No. 2	No. 3
Ground Truth	Peri-endo Combined Lesion	Normal	Apical Lesion
Validation	Peri-endo Combined Lesion	Normal	Apical Lesion
Accuracy	99.94%	60.47%	85.02%

**Table 8:** CNN validation results after padding.

Method	Metrics	AlexNet	Places365-GoogLeNet	VGG16	ResNet50	GoogLeNet	ConvNeXtv2
Original	Accuracy	80.95%	79.19%	71.43%	71.43%	80.95%	76.19%
	Training time	54 s	1 m 20 s	26 s	1 m 19 s	1 m 19 s	6 m 4 s
After padding	Accuracy	83.33%	80.95%	80.95%	73.81%	85.71%	85.71%
	Training time	48 s	1 m 29 s	24 s	1 m 19 s	1 m 17 s	5 m 46 s

**Table 9:** CNN validation results after data enhancement.

Method	Metrics	AlexNet	Places365-GoogLeNet	VGG16	ResNet50	GoogLeNet	ConvNeXtv2
Padding	Accuracy	83.33%	80.95%	80.95%	73.81%	85.71%	85.71%
	Training time	48 s	1 m 29 s	24 s	1 m 19 s	1 m 17 s	5 m 46 s
Padding + Enhancement	Accuracy	88.69%	88.69%	88.69%	88.69%	86.90%	87.50%
	Training time	2 m 49 s	5 m 17 s	1 m 2 s	4 m 33 s	5 m 26 s	21 m 58 s

**Table 10:** CNN validation results after expanding the cropping range.

Method	Metrics	AlexNet	Places365-GoogLeNet	VGG16	ResNet50	GoogLeNet	ConvNeXtv2
Original YOLOv8 cropping	Accuracy	88.69%	88.69%	88.69%	88.69%	86.90%	87.50%
	Training time	2 m 49 s	5 m 17 s	1 m 21 s	4 m 33 s	5 m 26 s	21 m 58 s
Expand x = 20 pixels, y = 0 pixels	Accuracy	91.67%	89.29%	90.48%	85.71%	89.29%	89.28%
	Training time	1 m 30 s	4 m 8 s	1 m 37 s	3 m 20 s	4 m 39 s	21 m 13 s
Expand x = 0 pixels, y = 40 pixels	Accuracy	88.10%	91.67%	90.48%	88.10%	88.69%	88.09%
	Training time	2 m 38 s	1 m 23 s	1 m 16 s	3 m 22 s	5 m 31 s	22 m 4 s
Expand x = 20 pixels, y = 40 pixels	Accuracy	89.29%	89.88%	88.10%	89.88%	88.10%	91.07%
	Training time	2 m 1 s	4 m 50 s	1 m 29 s	4 m 28 s	4 m 37 s	20 m 35 s

**Table 11:** CNN validation results after image enhancement.

Method	Metrics	AlexNet	Places365- GoogLeNet	VGG16	ResNet50	GoogLeNet	Conv NeXtv2
Original (padding, enhancement)	Accuracy	88.69%	88.69%	88.69%	88.69%	86.90%	87.50%
	Training time	2 m 49 s	5 m 17 s	1 m 21 s	4 m 33 s	5 m 26 s	21 m 58 s
Gaussian high-pass filter	Accuracy	92.26%	93..45%	91.67%	89.29%	92.26%	89.29%
	Training time	2 m 50 s	6 m 3 s	1 m 16 s	4 m 3 s	5 m 33 s	20 m 53 s
Adaptive histogram equalization	Accuracy	88.10%	92.86%	92.26%	91.07%	89.88%	95.23%
	Training time	50 s	1 m 16 s	1 m 21 s	4 m 1 s	5 m 14 s	55 m 40 s
Gaussian high-pass filter with adaptive histogram equalization	Accuracy	93.45%	91.07%	92.86%	90.48%	88.10%	93.45%
	Training time	2 m 24 s	5 m 37 s	1 m 30 s	3 m 55 s	5 m 48 s	22 m 2 s

**Table 12:** Confusion matrix for multi-class classification on validation set in CNN.

Disease		Actual		
		Normal	Apical Lesion	Peri-endo Combined Lesion
Predicted	Normal	56	4	0
	Apical Lesion	0	49	1
	Peri-endo Combined Lesion	0	3	55

**Table 13:** Comparison between different methods.

Method	The Best Model in this Study				Method in [8]	Method in [23]
Model	ConvNeXtv2				U-Net Model	Decision Tree
Disease	Normal	Apical Lesion	Peri-endo Combined Lesion	Total	Apical Lesion	Apical Lesion
Accuracy				95.23%	No data	No data
Precision	93.33%	98.00%	94.82%	95.38%	No data	No data
Recall	99.75%	87.50%	98.21%	95.23%	No data	No data
F1-Score	96.55%	92.45%	96.49%	95.16%	74.2%	89%

**Table 14:** YOLOv8 training set and validation set instance quantity.

Disease	Training Set	Validation Set	Total
Normal	194	58	252
Apical Lesion	106	20	126
Peri-endo Combined Lesion	70	15	85
Total	370	93	463

**Table 15:** YOLOv8 validation results after data enhancement.

Method	Metrics	Normal	Apical Lesion	Peri-Endo Combined Lesion	Total
Original	mAP50	0.871	0.742	0.928	0.847
	Accuracy				75.00%
Original with data enhancement	mAP50	0.906	0.878	0.927	0.904
	Accuracy				84.70%

**Table 16:** YOLOv8 validation result after image processing.

Method	Metrics	Normal	Apical Lesion	Peri-Endo Combined Lesion	Total
Linear Transformation with Adaptive histogram equalization	mAP50	0.904	0.876	0.957	0.912
	Accuracy				85.16%
Flat-Field Correction with Adaptive histogram equalization	mAP50	0.888	0.857	0.971	0.905
	Accuracy				87.79%
Gaussian high-pass filter with Negative Film Effect	mAP50	0.918	0.913	0.923	0.918
	Accuracy				92.13%

**Table 17:** Confusion matrix for multi-class classification with YOLO detection.

Disease		Actual		
		Normal	Apical Lesion	Peri-endo Combined Lesion
Predicted	Normal	143	3	2
	Apical Lesion	6	55	5
	Peri-endo Combined Lesion	2	2	36

**Table 18:** YOLOv8 model compared to models in other papers.

Method	The Best Models of this Study Method				Method in [24]	Method in [25]
Model	YOLOv8				YOLOv5x	YOLOv3 Darknet
Disease	Normal	Apical Lesion	Peri-endo Combined Lesion	Total	Apical Lesion	Apical Lesion
Accuracy				92.13%	No data	No data
Precision	69.3%	91%	86.4%	82.2%	83%	56%
Recall	84.2%	95%	91.7%	90%	No data	98%
mAP50	0.918	0.913	0.923	0.918	0.88	No data
F1-Score	87.46%	80.13%	88.49%	85.92%	87%	71%

## Data Availability

The data presented in this study are available in this article.
